# Dual-Energy Computed Tomography Versus Conventional Magnetic Resonance In-Phase/Opposed-Phase Imaging for Liver Fat Fraction Quantification: A Retrospective Cohort Study

**DOI:** 10.3390/diagnostics16193260

**Published:** 2026-10-08

**Authors:** Jing Hong Loo, Shi Min Yeow, Kang Ren Yong, Harvard Zhi Rui Lee, Sze Ying Yee, Srinivas Anandswaroop Uppaluri, Syed Aftab, Sandeep Halagatti Venkatesh, Freda Jawan, Shawn Shi Xian Kok, Praveen M. Yogendra, Min On Tan

**Affiliations:** 1 Department of Radiology, Sengkang General Hospital, Singapore 544886, Singapore; 2 Parkway Radiology, Singapore 228510, Singapore; 3 Department of Radiology, Eastern General Hospital, Singapore 168582, Singapore

**Keywords:** dual-energy CT, multi-material decomposition, MRI Dixon, in-phase imaging, opposed-phase imaging, hepatic steatosis

## Abstract

**Background/Objectives**: Multiple imaging modalities are available for non-invasive hepatic fat quantification. We performed a hypothesis-generating methods-comparison study to compare liver fat fraction (FF) measurements obtained using magnetic resonance imaging (MRI) in-phase/opposed-phase (IP/OP) with dual-energy computed tomography (DECT) multi-material decomposition (MMD) techniques. **Methods**: This retrospective method-comparison study included 45 patients who underwent abdominal DECT and MRI. Corresponding 2-cm^2^ regions of interest were placed in hepatic segments VI and VII. DECT and MRI fat fraction measurements were compared using Spearman’s correlation and Bland–Altman and Passing–Bablok analyses. Exploratory receiver operating characteristic area under the curve (ROC AUC) analyses evaluated the ability of DECT fat fraction measurements to discriminate MRI-defined steatosis thresholds. **Results**: DECT FF was positively associated with MRI FF in segment VI (Spearman’s ρ = 0.50, *p* < 0.001) and segment VII (ρ = 0.57, *p* < 0.001). Bland–Altman analysis showed that DECT measurements were lower than MRI measurements on average, with mean biases (MRI—DECT) of 3.39% and 2.57% for segments VI and VII respectively, accompanied by wide limits of agreement. Passing–Bablok analysis showed no statistically significant proportional or constant bias. The ability of DECT FF to classify MRI-defined steatosis improved with increasing disease severity, with AUCs of 0.77 for mild steatosis and 0.88-0.99 for moderate-to-severe steatosis. **Conclusions**: This preliminary method-comparison study demonstrates an association between DECT and MRI fat fraction measurements and supports the feasibility of DECT for opportunistic hepatic fat quantification. Although exploratory AUC analyses provide preliminary estimates for future validation studies, substantial inter-modality variability currently limits definitive clinical application.

## 1. Introduction

An accurate assessment of hepatic steatosis is essential for diagnosing and monitoring metabolic dysfunction-associated steatotic liver disease (MASLD), grading treatment response, and predicting cardiometabolic risk [1]. Historically, liver biopsy served as the reference standard, but its clinical utility is limited by its invasive nature, potential for procedural complications, and potential sampling variability [2]. Consequently, non-invasive imaging modalities have emerged as integral tools in both clinical practice and research trials.

Ultrasound (US) remains an accessible and relatively low-cost method for hepatic steatosis assessment. Qualitative US assessment is based on the evaluation of sonographic patterns like liver echogenicity and portal vein conspicuity. Quantitative assessment involves tissue attenuation imaging, the backscatter coefficient and controlled attenuation parameter [3]. However, these methods are affected by concurrent liver pathologies such as hepatitis or fibrosis, and suffer from inter-operator variability and low accuracy in obese patients [4]. Similarly, conventional computed tomography (CT) offers broad clinical utility. Hepatic fat quantification may be assessed using the absolute attenuation value of liver parenchyma or by the relative attenuation difference between liver parenchyma and spleen. Dual-energy computed tomography (DECT) has emerged as a promising alternative for hepatic evaluation. While single-energy CT relies on non-specific attenuation measurements that may be confounded by co-existing pathologies or intravenous contrast timing [5], DECT leverages a multi-material decomposition (MMD) algorithm to mathematically isolate tissue components [6]. By evaluating image voxels against predefined material pairs or triplets, MMD extracts an objective fat fraction (FF) from standard clinical datasets. This offers advantages over conventional attenuation measurements because overlapping density values between fat, fibrosis, and blood vessels limit the reliability of Hounsfield-based quantification alone.

Among magnetic resonance techniques, chemical-shift-encoded (CSE) magnetic resonance imaging (MRI) with proton density fat fraction (PDFF) mapping has been established as a quantitative reference method for fat assessment. MRI-PDFF utilizes specialized, multi-echo pulse sequences and advanced post-processing software to correct for T1, T2*, and spectral effects [7]. However, because MRI-PDFF requires specialized acquisitions that are not universally integrated into standard workflows, routine abdominal MRI protocols more frequently rely on traditional dual-echo in-phase and opposed-phase (IP/OP) imaging. In a recent prospective study, Hupfeld et al. explored the correlation of different MRI sequences for hepatic fat fraction quantification against a reference standard of histopathological examination. Both MRI IP/OP imaging and multi-gradient echo imaging showed a strong correlation with biopsy FF [8].

These operational and algorithmic differences directly influence the diagnostic performance and practical utility of each modality. Conventional MRI IP/OP allows subjective evaluation of hepatic steatosis, but its quantitative metrics can be confounded by severe hepatic iron overload [9]. The potential advantage of DECT MMD lies in its ability to be integrated into routine contrast-enhanced protocols, allowing for retrospective fat quantification via virtual unenhancement processing. Recognizing the practical need to benchmark these more readily available clinical techniques against one another, we conducted a hypothesis-generating, method-comparison study in our institution to compare liver FF measurements from MRI IP/OP with DECT MMD. We aimed to provide exploratory analyses and preliminary comparative thresholds for opportunistic steatosis assessment.

## 2. Materials and Methods

### 2.1. Study Population

According to local institutional guidelines, ethics approval and informed consent were not required because the study used retrospectively collected data that had been de-identified before investigator review. The cohort comprised all patients who underwent both abdominal CT and MRI at Sengkang General Hospital, Singapore, between 2023 and 2025. Patients with motion artefacts precluding reliable region-of-interest (ROI) placement, known hepatic malignancy, or focal hepatic lesions within the target segments were excluded. Clinical trial number is not applicable.

### 2.2. MRI Protocol

All MRI examinations were performed using a 3 Tesla scanner (Siemens MAGNETOM Vida; Siemens Healthineers, Erlangen, Germany). T1-weighted volumetric interpolated breath-hold examination (VIBE) Dixon sequences were acquired for the upper abdomen, incorporating both in-phase (IP) and opposed-phase (OP) imaging, alongside the generation of fat- and water-selective maps. MRI acquisition parameters for IP/OP were as follows: breath-hold lasting 15 s, repetition time (TR) of 3.97 ms; out-of-phase echo time (TE) of 1.29 ms, in-phase TE of 2.52 ms, flip angle of 9°; field of view (FOV) of 380 × 309 mm; matrix of 320 × 256; parallel imaging factor (GRAPPA) of 2; intersection gap of 0 mm; number of excitations (NEX) equal to 1; voxel size of 1.2 × 1.2 × 2.5 mm^3^; and slice thickness = 2.5 mm. These parameters align with vendor guidance for reproducible single-breath-hold Dixon acquisitions.

### 2.3. CT Protocol

All dual-energy CT examinations were performed using a third-generation dual-source scanner (Siemens SOMATOM Force, Siemens Healthineers, Forchheim, Germany). Following scout acquisition, images were obtained with the patient in a supine position and in the craniocaudal scanning direction. Data acquisition commenced 80 s after the administration of 1 mL/kg of a non-ionic iodine contrast agent at 3.5 mL/s (Iohexol 350 mg I/mL, Omnipaque 350), followed by a 30 mL saline flush. The CT acquisition parameters were as follows: simultaneous dataset acquisition at 100/Sn150 kVp; reference mAs at 190/95; collimation of 2 × 128 × 0.6 mm; scanning FOV of 350 mm; the spiral scanning mode; and rotation time of 0.5 s/cycle; pitch 0.6. Images were reconstructed in the axial, sagittal and coronal planes using a soft-tissue kernel (Br40) and an iterative reconstruction technique (ADMIRE, strength 3) at a slice thickness of 3 mm, reconstructed section interval of 3 mm, display FOV of 300 mm, and matrix of 512 × 512. Dose optimization was performed via automatic exposure control (CARE Dose 4D, Siemens Healthineers).

### 2.4. Image Evaluation

**Figure 1:**
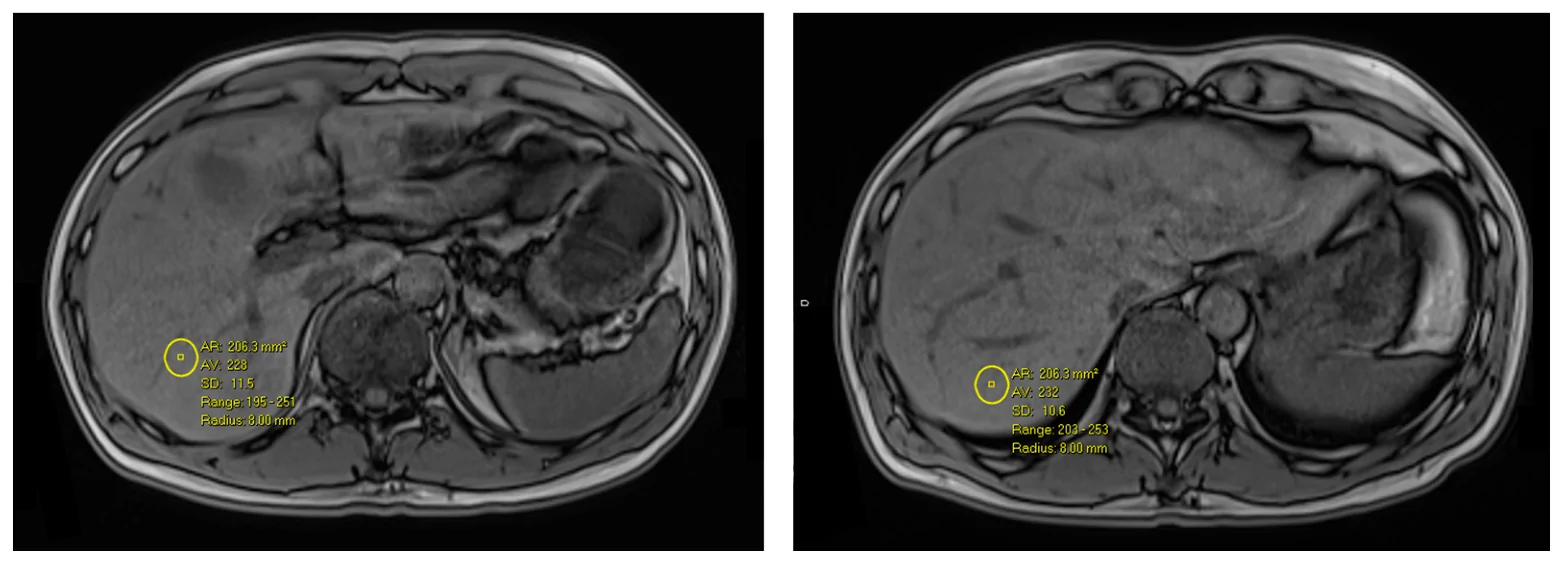
Representative regions of interest (ROIs) placed in hepatic segment VI (**left** image) and segment VII (**right** image) on MRI in-phase/out-of-phase imaging, avoiding major hepatic vasculature.

Abdominal MRI images were analysed by one author (HZRL) who manually drew two ROIs of size 2 cm^2^ in hepatic segments VI and VII (Figure 1). These segments were chosen due to their large homogenous volume and standard vascular inflow, reduced susceptibility to cardiac pulsation and respiratory motion, and avoidance of focal fat sparing or infiltration [10]. The ROIs were selected in liver parenchyma not containing large hepatic vessels, biliary structures, or focal lesions [11]. In-phase and opposed-phase liver signal intensities in hepatic segments VI and VII were processed via a web-based radiology tool. The formula applied was $\mathrm{Fat}\;\mathrm{fraction}=100\times \frac{\mathrm{liver}\;\mathrm{SIIP}-\mathrm{liver}\;\mathrm{SIOP}}{2\times \mathrm{liver}\;\mathrm{SIIP}}$, where SIIP is the in-phase signal intensity and SIOP is the opposed-phase signal intensity (Phillip Cheng Hepatic Fat MRI Calculator; available at https://pcheng.org/calc/hepatic_fat_mri.html; accessed on 1 June 2026). This tool is based on standard Dixon chemical shift imaging principles [12].

Dual-energy CT post-processing was performed using the Dual Energy Liver Virtual Non-Contrast (Liver VNC) application in Syngo.via VB80E (Siemens Healthineers, Erlangen, Germany). The application employs a three-material decomposition model incorporating soft tissue, fat, and iodine. Using the dual-energy attenuation characteristics of these materials, a virtual non-contrast (VNC) image is generated from the contrast-enhanced acquisition. Quantitative fat maps are subsequently derived from the VNC dataset by linear rescaling with application of a weak noise-reduction filter. Fat calibration was based on predefined reference materials in which soft tissue corresponded to 0% fat and fat corresponded to 100% fat. For the SOMATOM Force 100/Sn150 kVp acquisition protocol, the material definitions were specified by attenuation values of 57 and 56 HU for soft tissue and −105 and −84 HU for fat in the low- and high-energy datasets, respectively. Default vendor calibration settings were used throughout the study, and no additional user-defined calibration was performed.

**Figure 2:**
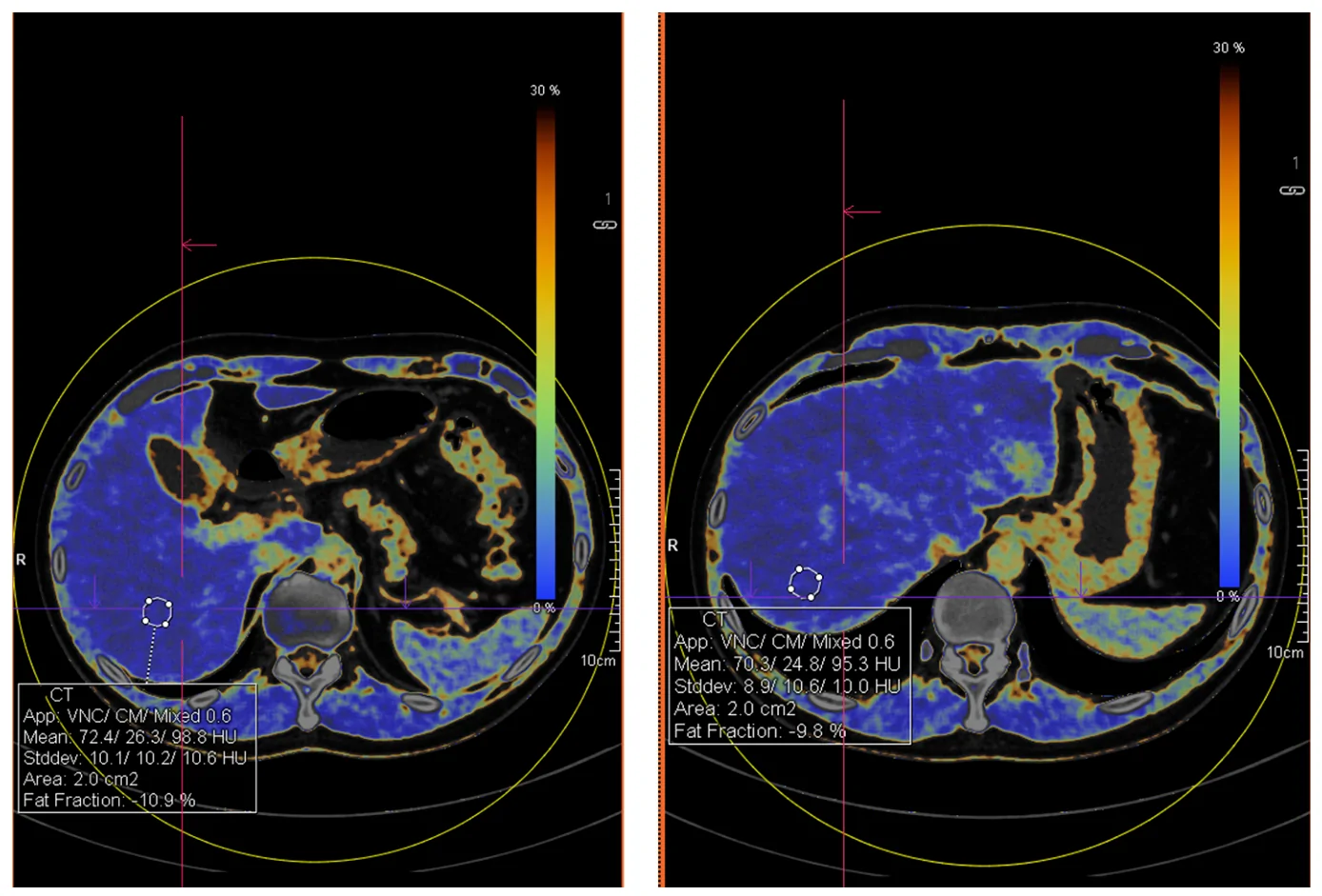
Corresponding ROI placement on dual-energy computed tomography hepatic fat map generated by multi-material decomposition (Syngo.Via), matched to MRI ROIs.

The same author (HZRL) manually placed matching ROIs on the Syngo.Via-generated quantitative fat maps (Figure 2). All ROIs were located fully within the dual-energy FOV. The ROI locations and measurements were reviewed by another author (JHL) to ensure anatomical correspondence between DECT and MRI images and adherence to predefined ROI placement criteria. In cases where disagreement regarding ROI location occurred, the images were reviewed jointly with a senior consultant radiologist (MOT), and a consensus measurement was used for the final analysis.

### 2.5. Statistical Analysis

An a priori sample size estimation was conducted using R software (version 4.6.1) to ensure adequate statistical power across all primary outcomes. With a two-sided significance level of 0.05 and a target power (1–β) of 0.80, the analysis established that a sample size of 33 participants would be required for an expected Spearman’s ρ of 0.50.

Negative fat fractions may arise due to random image noise, reconstruction variations and T2* decay in areas where the true fat content is near zero [13,14]. We chose to maintain all negative values in the analyses to prevent artificial upward bias and preserve the statistical validity of the dataset.

Measurements from segments VI and VII were analysed separately rather than averaged. Averaging values from only two segments may not accurately represent whole-liver fat content. Comprehensive averaging would require ROI sampling across multiple liver segments, which was beyond the scope of the present study.

Continuous variables were expressed as the median (interquartile range, IQR). The primary outcome was the association between DECT FF and MRI IP/OP FF, assessed using Spearman’s rank correlation analysis and supplemented by scatter plots with linear regression lines. Bland–Altman plots were subsequently generated to evaluate systematic measurement bias by calculating the mean difference and 95% limits of agreement between the techniques. Passing–Bablok regression analysis was performed to assess proportional and constant bias between DECT and MRI measurements. Receiver operating characteristic area under the curve (ROC AUC) analysis was used to evaluate the performance of DECT in identifying hepatic steatosis relative to MRI FF quantification. The efficacy of DECT for categorizing steatosis severity was evaluated across three thresholds: mild (MRI FF ≥ 5%), moderate (MRI FF ≥ 15%) and severe (MRI FF ≥ 25%). These exploratory thresholds were derived from previously reported MRI-PDFF categories [15,16], as validated severity cutoffs for MRI IP/OP FF measurements are not currently established. Optimal DECT cutoff values were selected using the Youden index. Sensitivity, specificity, positive predictive value, and negative predictive value were reported with 95% confidence intervals estimated using the Wilson method.

Sensitivity analyses were conducted to assess the robustness of the primary outcome. The primary Spearman correlation analysis was repeated using patient-level average fat fraction values derived from segments VI and VII. The potential effect modification of the time interval between examinations on the inter-modality association was assessed using a multiple linear regression model with DECT FF as the dependent variable, and MRI FF, scan interval, and their interaction term as independent variables. Statistical significance was defined as a two-sided *p*-value < 0.05. All statistical computations were performed using R software, version 4.6.1.

## 3. Results

### 3.1. Patient Characteristics

A total of 51 patients who underwent both abdominal CT and MRI examinations between 2023 and 2025 were initially identified for screening. Six patients were excluded, including three who underwent CT imaging on a different vendor platform (GE Healthcare) and three with inadequate image quality due to motion artefacts precluding reliable ROI placement. The final study cohort comprised 45 patients, including 20 males (44.4%) and 25 females (55.6%). The median age was 40 (36–56) years old. These patients underwent CT and MRI body imaging for routine clinical indications unrelated to hepatic steatosis. The median CT FF was 4.26% (−1.00–8.80%) for segment VI and 4.80% (0.66–11.90%) for segment VII. The time interval between CT and MRI examinations was 123 (86–264) days. The median MRI FF was 5.30% (0.80–9.50%) for segment VI and 8.71% (2.10–12.10%) for segment VII. Of the 45 patients, 20 (44.4%) had no steatosis (average MRI FF < 5%), 18 (40.0%) had mild steatosis (average MRI FF ≥ 5%), 3 (6.67%) had moderate steatosis (MRI FF ≥ 15%), and 4 (8.89%) had severe steatosis (average MRI FF ≥ 25%).

Negative fat fraction values were observed in both MRI and DECT measurements. In segment VI, negative values were present in 9/45 (20.0%) MRI measurements and 15/45 (33.3%) DECT measurements. In segment VII, negative values were present in 8/45 (17.8%) MRI measurements and 11/45 (24.4%) DECT measurements. Among the negative measurements, the median (IQR) values were −0.90% (−1.70% to −0.60%) for MRI segment VI, −1.65% (−2.15% to −0.11%) for MRI segment VII, −3.34% (−6.14% to −0.93%) for DECT segment VI, and −3.68% (−5.98% to −2.71%) for DECT segment VII.

### 3.2. Correlation Analysis

**Figure 3:**
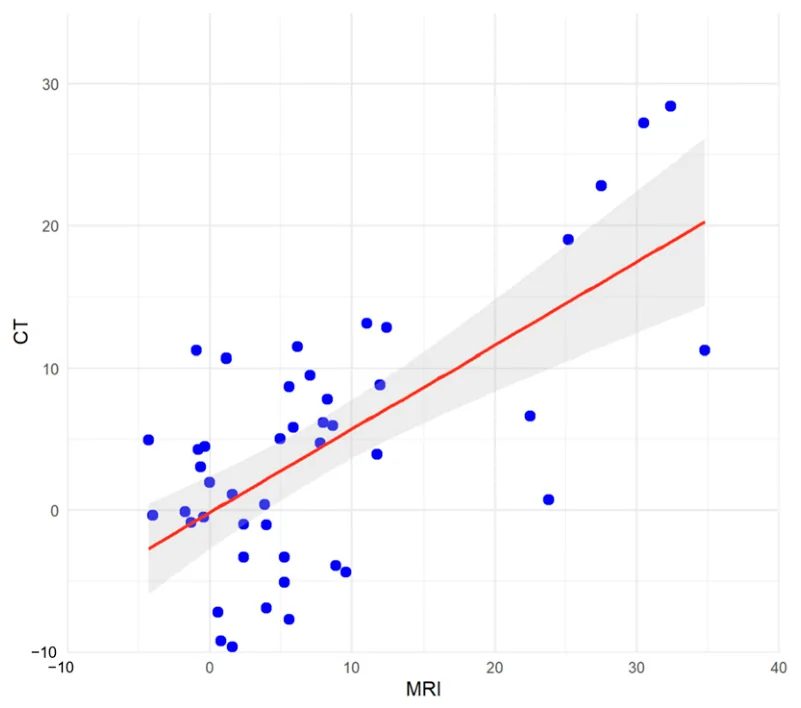
Spearman correlation scatter plot for segment VI: Computed tomography (CT) fat fraction versus magnetic resonance imaging (MRI) fat fraction (Spearman’s ρ = 0.50, *p* < 0.001). The red line indicates the fitted linear regression line, and the grey shaded area represents the 95% confidence interval of the regression estimate.

**Figure 4:**
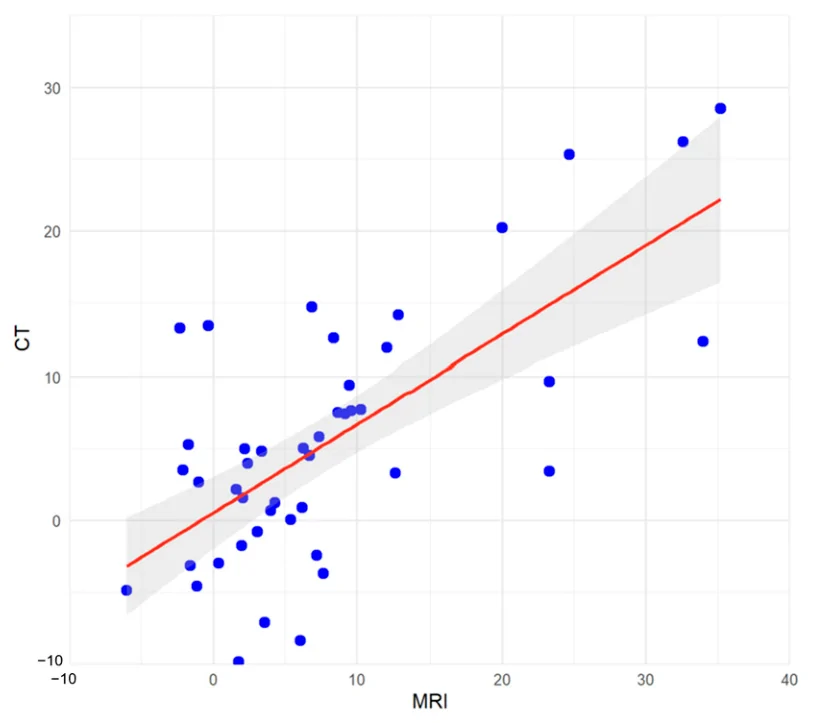
Spearman correlation scatter plot for segment VII: Computed tomography (CT) fat fraction versus magnetic resonance imaging (MRI) fat fraction (Spearman’s ρ = 0.57, *p* < 0.001). The red line indicates the fitted linear regression line, and the grey shaded area represents the 95% confidence interval of the regression estimate.

The relationship between DECT FF and MRI FF was examined using Spearman’s rank correlation. DECT and MRI FF values demonstrated a statistically significant positive correlation for both segment VI (Spearman ρ = 0.50; *p* < 0.001) (Figure 3) and segment VII (Spearman’s ρ = 0.57; *p* < 0.001) (Figure 4).

### 3.3. Agreement Analysis

**Figure 5:**
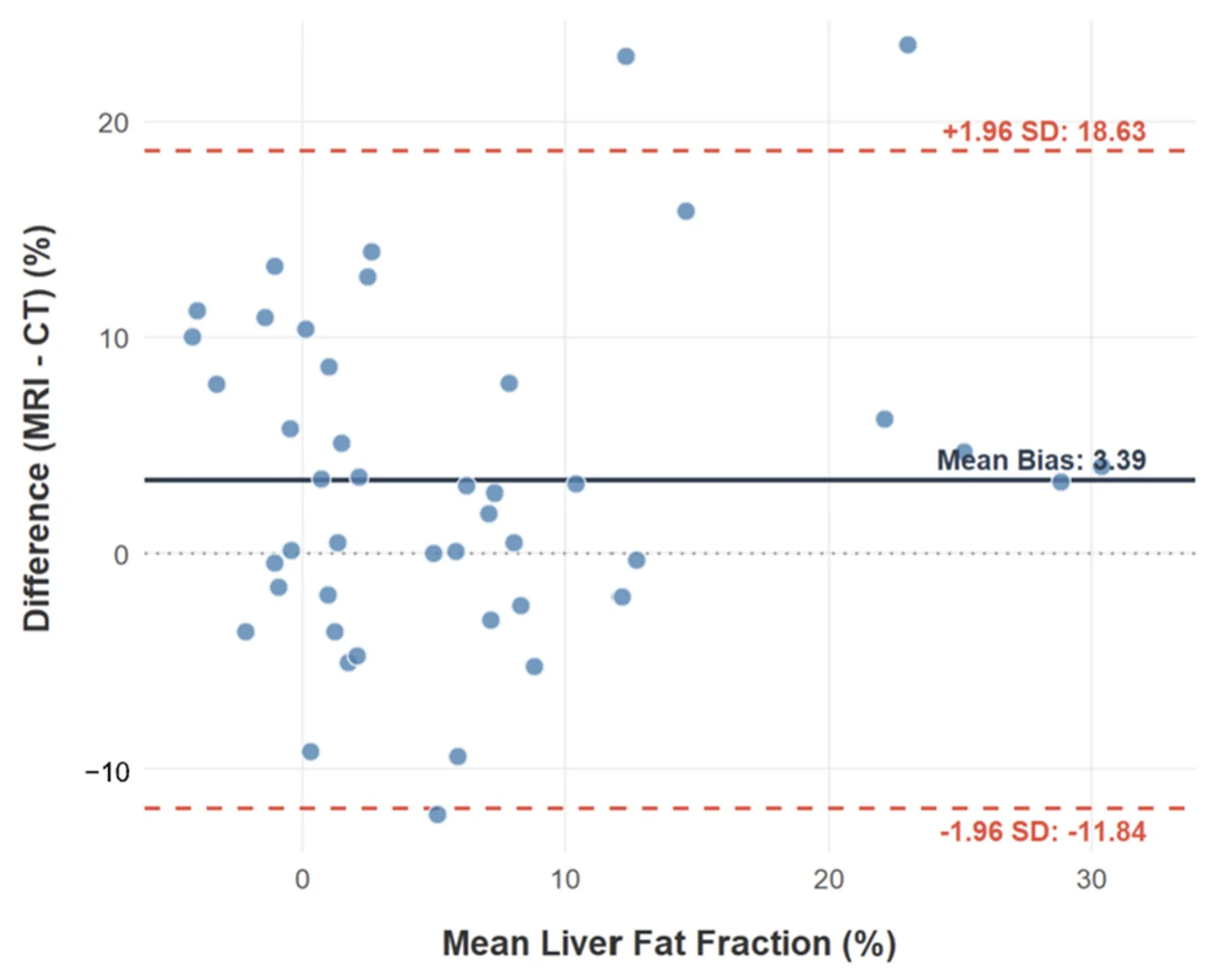
Bland–Altman plot for segment VI showing mean bias 3.39% (95% CI: 1.06% to 5.73%) and 95% limits of agreement (−11.84% to 18.63%). MRI = magnetic resonance imaging; CT = computed tomography; SD = standard deviation.

**Figure 6:**
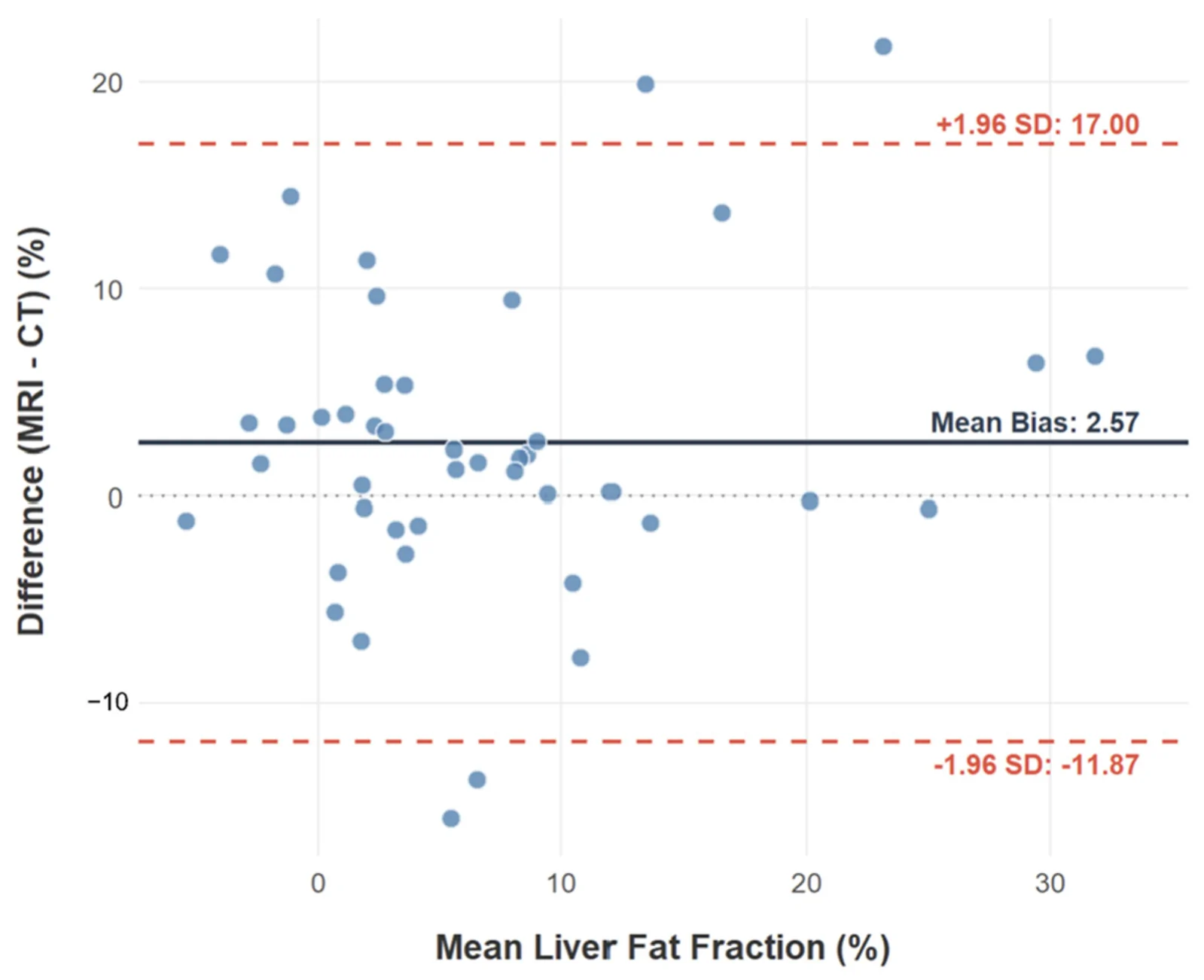
Bland–Altman plot for segment VII showing mean bias 2.57% (95% CI: 0.35% to 4.78%) and 95% limits of agreement (−11.87% to 17.00%). MRI = magnetic resonance imaging; CT = computed tomography; SD = standard deviation.

Bland–Altman analysis was performed to assess systematic bias and the 95% limits of agreement between DECT and MRI fat fraction measurements. For segment VI, the mean bias (MRI—CT) was 3.39% (95% confidence interval (CI): 1.06% to 5.73%). The lower and upper limits of agreement were −11.84% (95% CI: −15.89% to −7.80%) and 18.63% (95% CI: 14.58% to 22.67%) respectively (Figure 5). For segment VII, the mean bias was 2.57% (95% CI: 0.35% to 4.78%), with corresponding lower and upper limits of agreement of −11.87% (95% CI: −15.70% to −8.04%) and 17.00% (95% CI: 13.17% to 20.84%) respectively (Figure 6). These findings demonstrate systematic inter-modality differences in fat fraction measurements across both segments, with DECT yielding lower fat fraction estimates than MRI on average.

**Figure 7:**
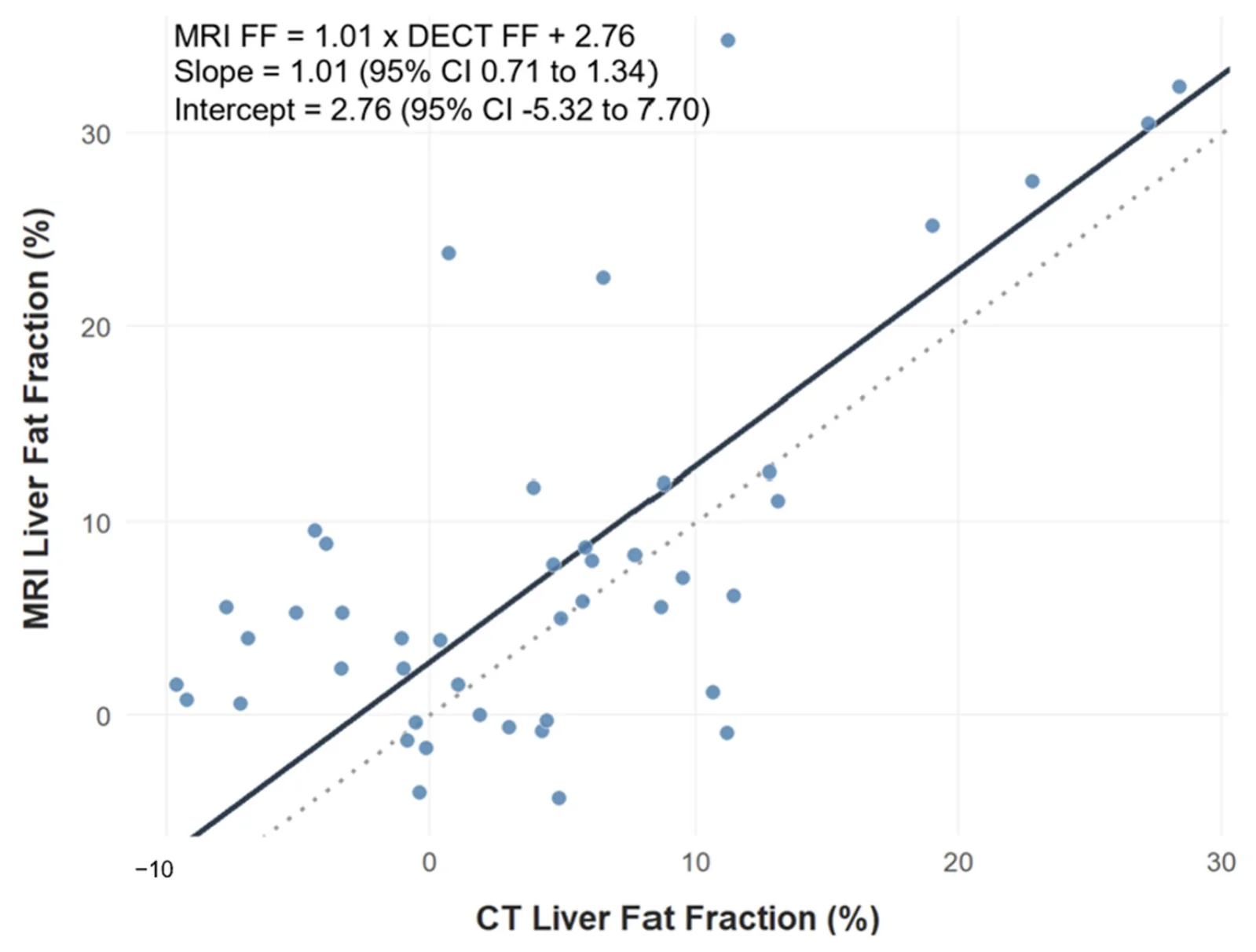
Passing–Bablok regression plot for segment VI. The solid black line represents the regression equation MRI FF = 1.01 × DECT FF + 2.76, with a slope of 1.01 (95% CI: 0.71 to 1.34) and an intercept of 2.76% (95% CI: −5.32% to 7.70%). The dotted line represents the line of identity (y = x). MRI = magnetic resonance imaging; CT = computed tomography; CI = confidence interval; FF = fat fraction.

**Figure 8:**
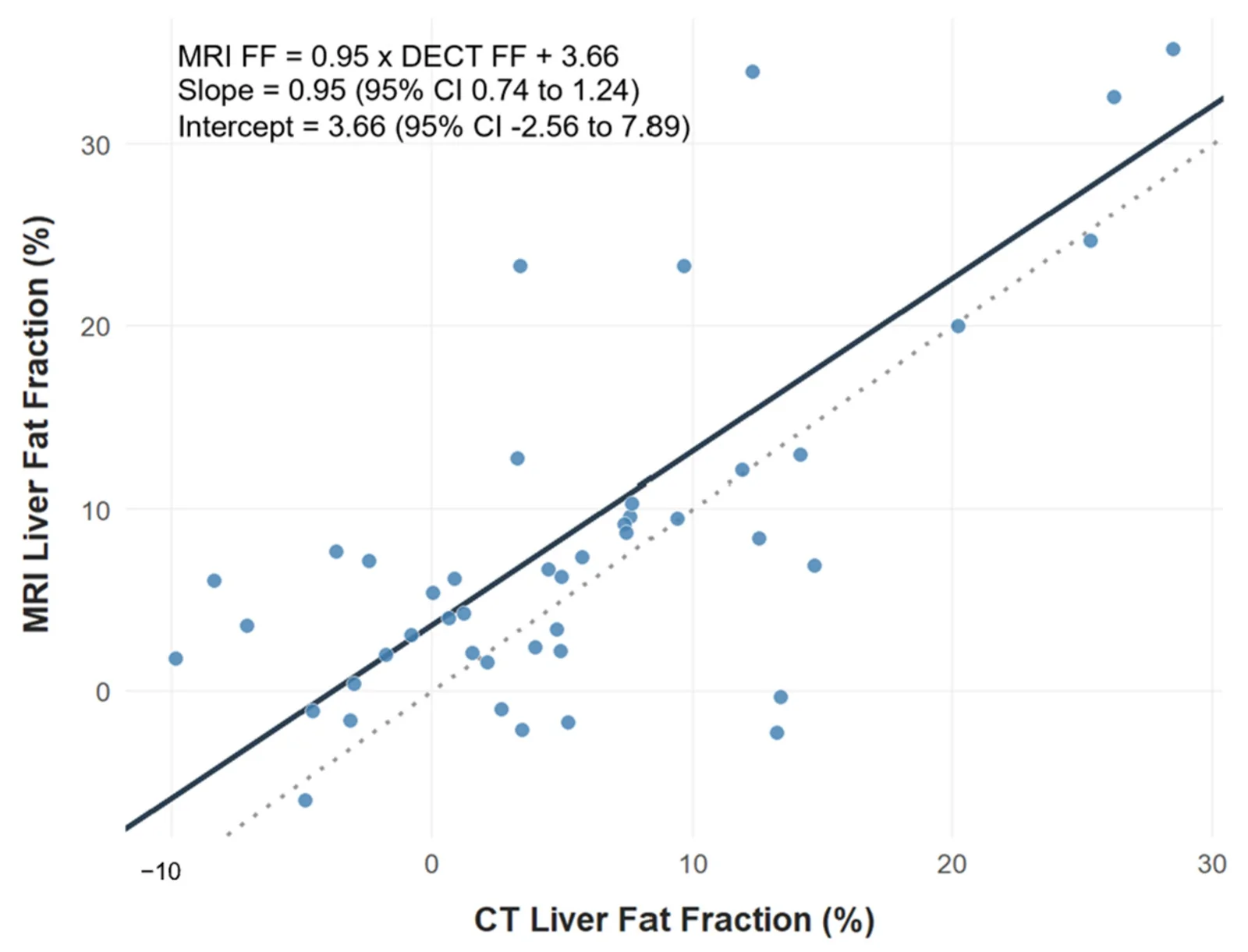
Passing–Bablok regression plot for segment VII. The solid black line represents the regression equation MRI FF = 0.95 × DECT FF + 3.66, with a slope of 0.95 (95% CI: 0.74 to 1.24) and an intercept of 3.66% (95% CI: −2.56% to 7.89%). The dotted line represents the line of identity (y = x). MRI = magnetic resonance imaging; CT = computed tomography; CI = confidence interval; FF = fat fraction.

Passing–Bablok regression analysis was performed to assess for constant and proportional bias between DECT and MRI FF measurements. For segment VI, the estimated regression equation was MRI FF = 1.01 × DECT FF + 2.76. The slope was 1.01 (95% CI: 0.71 to 1.34) and the intercept was 2.76% (95% CI: −5.32% to 7.70%) (Figure 7). For segment VII, the estimated regression equation was MRI FF = 0.95 × DECT FF + 3.66. The slope was 0.95 (95% CI: 0.74 to 1.24) and the intercept was 3.66% (95% CI: −2.56% to 7.89%) (Figure 8). As the confidence intervals for the slopes included 1 and the confidence intervals for the intercepts included 0 in both segments, there was no statistically significant evidence of proportional or constant bias between DECT and MRI measurements.

### 3.4. Exploratory Receiver Operating Characteristic Analysis

ROC analyses assessed the ability of DECT FF measurements to discriminate MRI IP/OP-defined steatosis categories using thresholds of ≥5%, ≥15%, and ≥25% for mild, moderate, and severe steatosis respectively (Table 1). These thresholds are defined independently for each liver segment. For mild steatosis, AUC values were 0.77 in both segments, with higher specificity (90%) but lower sensitivity (64–68%). Exploratory analyses for moderate steatosis demonstrated higher AUC values (0.88–0.89), with segment VII showing higher sensitivity than segment VI. The highest AUC values were observed for severe steatosis (0.94–0.99), with sensitivity reaching 100% in both liver segments.

### 3.5. Sensitivity Analysis

The potential influence of the interval between CT and MRI examinations on the observed association between modalities was assessed as a sensitivity analysis. No statistically significant interaction was identified for either segment VI (β = 0.0002, 95% CI: −0.001 to 0.001; t = 0.22, *p* = 0.83) or segment VII (β = 0.0002, 95% CI: −0.001 to 0.001; t = 0.42, *p* = 0.67). These findings indicate no evidence of significant effect modification by scan interval on the association between modalities in either liver segment.

**Figure 9:**
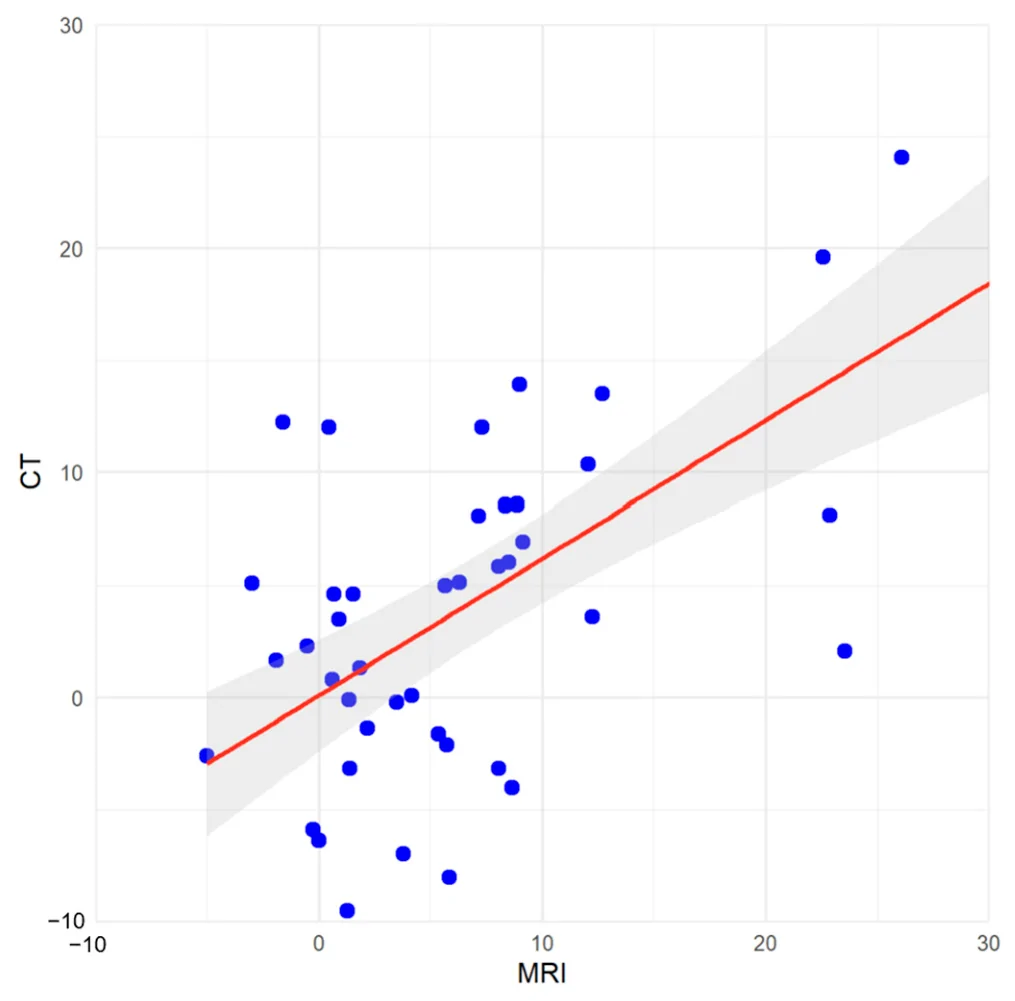
Spearman correlation scatter plot for patient-level average fat fraction: Computed tomography (CT) fat fraction versus magnetic resonance imaging (MRI) fat fraction (Spearman’s ρ = 0.53, *p* < 0.001). The red line indicates the fitted linear regression line, and the grey shaded area represents the 95% confidence interval of the regression estimate.

As an additional sensitivity analysis, patient-level average fat fraction values were calculated by averaging measurements from segments VI and VII. Repeating the primary Spearman correlation analysis using these averaged values demonstrated a positive association between DECT and MRI IP/OP fat fraction measurements (Spearman ρ = 0.53, *p* < 0.001) (Figure 9). This finding supports the primary results and suggests that the observed inter-modality relationship was not dependent on segment-specific measurements alone.

## 4. Discussion

The present study demonstrates a statistically significant, moderate correlation between DECT MMD and MRI IP/OP FF (Spearman’s ρ = 0.50–0.57). Several studies have evaluated the role of DECT as a precise modality for hepatic fat quantification. Hong et al. evaluated a specialized Multi-Material Decomposition (MMD) algorithm utilizing a low-dose, non-contrast DECT protocol and demonstrated strong correlation with MRI-PDFF (r = 0.76) [17]. Furthermore, Zhang et al. found that material decomposition fat quantification outperformed conventional attenuation-based approaches, with lipid map-derived fat fraction showing closer agreement with MRI-PDFF than the liver–spleen (L/S) attenuation ratio [18]. While prior investigation in this domain has focused on validating DECT against MRI-PDFF, our study explicitly evaluates its correlation with conventional two-point Dixon IP/OP imaging. This distinction is critical because, although PDFF is recognized as a highly controlled quantitative reference standard, traditional IP/OP signal intensity metrics remain far more ubiquitous in routine diagnostic radiology workflows. The observed correlations indicate that higher DECT-derived fat fraction values generally corresponded to higher MRI-derived fat fraction values, supporting the ability of DECT to reflect overall trends in hepatic fat accumulation within the cohort.

A key finding of our study was that DECT and MRI IP/OP yielded systematically different fat fraction measurements. Bland–Altman analysis demonstrated positive mean biases (MRI—CT) of 3.39% for segment VI and 2.57% for segment VII, indicating that DECT produced lower fat fraction estimates than MRI on average. Passing–Bablok regression yielded positive intercept estimates in both segments, suggesting the same directional pattern. However, the confidence intervals for the intercepts included zero, indicating that a statistically significant constant bias could not be established. The observed difference between modalities is likely multifactorial: DECT FF estimates are influenced by the underlying material decomposition algorithm, calibration parameters, contrast enhancement, and image artefacts (partial-volume effects and beam-hardening artefacts) [19,20], whereas MRI IP/OP imaging lacks confounder correction for T1, T2* and spectral effects [21]. However, the Bland–Altman limits of agreement were broad, spanning approximately 30 percentage points in both segments. This range is substantial relative to the steatosis grading thresholds used in this study (5%, 15%, and 25%). Although Passing–Bablok regression did not demonstrate significant proportional or constant bias, the relatively wide confidence intervals of the slope and intercept estimates and the scatter of data indicate considerable measurement variability between modalities. As a result, the present findings support an overall association between DECT and MRI measurements but do not support their direct interchangeability for individual patient classification or longitudinal monitoring.

The exploratory ROC analyses suggested that classification concordance between DECT and MRI increased with steatosis severity. AUC values were lower for mild steatosis (0.77; sensitivity 64–68%) and higher for moderate-to-severe steatosis (0.88–0.99; specificity 84–93%). This pattern is consistent with the broader literature suggesting that DECT-based fat quantification is better suited for detecting clinically significant (moderate-to-severe) steatosis rather than early or mild disease [15,22]. However, the number of patients with moderate or severe steatosis was small. Consequently, the high AUC estimates and Youden-derived cutoffs should be interpreted cautiously, as they may be unstable. The similar or overlapping cutoff values identified for moderate and severe steatosis suggest a lack of precision. Therefore, these findings should be regarded as exploratory and hypothesis-generating rather than clinically applicable thresholds. Nevertheless, they may provide useful preliminary estimates for future validation studies in larger cohorts.

Sensitivity analyses did not identify evidence of significant effect modification by scan interval on the association between DECT and MRI FF measurements. This is reassuring for retrospective study designs in which scan pairing is opportunistic and may span weeks to months. However, hepatic steatosis is a dynamic process sensitive to short-term changes in diet, weight and metabolic status [23]. The relatively long and variable interval between CT and MRI examinations may result in individual-level fluctuations. This remained a limitation of our study.

Various strategies have been described for hepatic fat quantification, ranging from single-ROI, multi-ROI sampling, automated segmentation, and whole-liver volumetric approaches. Single-ROI approaches are simple and time-efficient but may be susceptible to sampling bias when steatosis is heterogeneously distributed [24]. Multi-ROI techniques provide a more representative estimate of liver fat content but require additional measurement time and can be technically challenging in segments II, III, and IV because of their smaller size and proximity to vascular and biliary structures [25]. Automated and whole-liver volumetric approaches further reduce operator dependency and have demonstrated high accuracy, although slight overestimation compared with manual ROI measurements has been reported [26,27]. In the present study, ROIs were placed in segments VI and VII because these segments are relatively large, less affected by cardiac motion, and generally allow reproducible placement of sufficiently sized ROIs while avoiding major vessels, bile ducts, and edge artefacts. Although this approach may not fully represent whole-liver fat content, the use of corresponding ROIs in both DECT and MRI ensured anatomical consistency for inter-modality comparison. Furthermore, sensitivity analysis using averaged measurements from segments VI and VII yielded results similar to the primary analyses, suggesting that the observed DECT-MRI association was not solely driven by a single ROI location. However, this should not be considered equivalent to established multi-segment assessment strategies. Future studies incorporating automated multi-segment or whole-liver volumetric analyses may further improve the representativeness and reproducibility of hepatic fat quantification.

Our study has limitations inherent to retrospective single-institution designs. Firstly, we had a small sample size (N = 45) with few patients in the moderate and severe steatosis category. While this was sufficient for analysis of the primary outcome involving the entire cohort, the study was underpowered for subgroup analysis such as ROC analysis. Secondly, this was a method-comparison study using MRI IP/OP imaging as the comparator rather than MRI-PDFF or histologic assessment. Although MRI IP/OP is widely available and commonly used in routine clinical practice, it does not incorporate the T1, T2*, and spectral confounder corrections that characterize PDFF. Consequently, the observed relationship between DECT and MRI in this study should be interpreted within the context of comparison to a pragmatic clinical imaging technique rather than a quantitative reference method. Furthermore, the exploratory ROC analyses used steatosis thresholds derived from previously reported MRI-PDFF categories, as validated severity thresholds for MRI IP/OP fat fraction measurements have not been established. Thirdly, information on chronic liver disease, iron overload, treatment changes, and weight changes between imaging examinations was not consistently available because of the retrospective design. This may have introduced unmeasured variability and residual confounding into our analysis. Fourth, the retrospective design resulted in variable time intervals between DECT and MRI examinations and individual-level fluctuations may have introduced measurement variability in our study. Lastly, formal assessment of measurement reproducibility was not performed. ROI placement and measurements were undertaken by a single reader and subsequently reviewed for anatomical correspondence by a second reader. Consequently, inter-reader and intra-reader agreement were not formally evaluated, and the extent to which measurement variability may have influenced the reported fat fraction estimates remains uncertain.

## 5. Conclusions

This preliminary method-comparison study demonstrated a moderate positive association between DECT MMD and MRI IP/OP fat fraction measurements, indicating that higher DECT-derived fat fraction values generally corresponded to higher MRI-derived fat fraction values. Although exploratory ROC analyses suggested improved discrimination with increasing steatosis severity, these findings should be interpreted cautiously given the wide limits of agreement, small number of patients with moderate and severe steatosis, and exploratory nature of the cutoff analyses. The proposed cutoffs should be considered preliminary and hypothesis-generating and will require external validation in larger prospective cohorts before any clinical application. Therefore, the current data support feasibility and association rather than established clinical performance for hepatic fat assessment using DECT MMD. Future studies should evaluate DECT MMD using standardized acquisition protocols, multi-vendor validation, and comparisons with quantitative reference methods to better define its role in hepatic steatosis assessment.

## Figures and Tables

**Table 1:** Exploratory ROC analysis of DECT fat fraction for MRI-defined steatosis severity categories. Values in parentheses indicate 95% confidence intervals.

Segment	Reference	AUC	Cutoff	Sensitivity	Specificity	PPV	NPV	Youden J
VI	MRI-FF ≥ 5%	0.77 (0.63–0.91)	4.96	0.68 (0.48–0.83)	0.90 (0.70–0.97)	0.89 (0.69–0.97)	0.69 (0.50–0.84)	0.58
VI	MRI-FF ≥ 15%	0.88 (0.72–1.00)	11.21	0.71 (0.36–0.92)	0.92 (0.79–0.97)	0.63 (0.31–0.86)	0.95 (0.82–0.99)	0.64
VI	MRI-FF ≥ 25%	0.99 (0.95–1.00)	11.21	1.00 (0.57–1.00)	0.93 (0.80–0.97)	0.63 (0.31–0.86)	1.00 (0.91–1.00)	0.93
VII	MRI-FF ≥ 5%	0.77 (0.63–0.91)	5.53	0.64 (0.45–0.80)	0.90 (0.70–0.97)	0.89 (0.67–0.97)	0.67 (0.48–0.81)	0.54
VII	MRI-FF ≥ 15%	0.89 (0.74–1.00)	9.52	0.86 (0.49–0.97)	0.84 (0.70–0.93)	0.50 (0.25-0.75)	0.97 (0.85-0.99)	0.70
VII	MRI-FF ≥ 25%	0.94 (0.83–1.00)	12.09	1.00 (0.44–1.00)	0.83 (0.69–0.92)	0.30 (0.11–0.60)	1.00 (0.90–1.00)	0.83

MRI-FF = magnetic resonance imaging derived fat fraction; AUC = area under the receiver operating characteristic curve; PPV = positive predictive value; NPV = negative predictive value; Youden J = Youden index.

## Data Availability

Data and associated analysis are held in a repository by the study authors. The data can be made available upon request.

## Abbreviations

The following abbreviations are used in this manuscript:

MASLD	Metabolic dysfunction-associated steatotic liver disease
MRI	Magnetic resonance imaging
IP/OP	In-phase/opposed-phase imaging
DECT	Dual-energy computed tomography
MMD	Multi-material decomposition
ROI	Region of interest
CI	Confidence interval
CSE	Chemical-shift encoded
PDFF	Proton density fat fraction
FF	Fat fraction
VIBE	Volumetric interpolated breath-hold examination
TR	Repetition time
TE	Echo time
FOV	Field of view
NEX	Number of excitations
IQR	Interquartile range
ROC AUC	Receiver operating characteristic area under the curve
PPV	Positive predictive value
NPV	Negative predictive value
SD	Standard deviation
